# Does Advanced Human Capital Structure Provide Positive Feedback on Public Health? Evidence From China

**DOI:** 10.3389/fpubh.2022.829716

**Published:** 2022-03-09

**Authors:** Tian-Hui Wang, Jin Lu

**Affiliations:** School of Economics, Qingdao University, Qingdao, China

**Keywords:** advanced human capital structure, public health, China, Grossman, panel threshold regression model

## Abstract

This paper explores the relationship of advanced human capital structure with public health applying the panel threshold regression model in China. The empirical results highlight that the advanced human capital structure has a non-linear single threshold effect on population health indicators. The health-promoting effect of advanced human capital structure is significantly weaker when exceeding the threshold. These asymmetric effects are strongly related to the response of China's health policies. The promotion effect of the advanced human capital structure on public health has significant heterogeneity in different regions. There is a single threshold value in the eastern and central regions, but the threshold value and facilitation effect are different. However, the western region has no threshold. The heterogeneity effects are caused by the different levels of advanced human capital structure. Governments should adopt appropriate public health policies according to the development characteristics of different regions.

## Introduction

The economy of China developed rapidly by virtue of policy dividends and factor price advantages, following the Reform and Opening-up Policy. At the same time, the health of the public is also showing an upward trend. According to data from the National Bureau of Statistics of China, the average life expectancy of residents rose from 67.8 years in 1981 to 74.8 years in 2010 and reached 77.0 years in 2018. In the process of continuous improvement of public health, education, which is a key element of human capital, has gradually transformed and upgraded from simple labor to advanced human capital. Among them, the development of higher education is the most important driving force. From 1949 to 2018, the gross enrollment rate of higher education in China increased from 0.26 to 48.1%, the total enrollment scale of colleges and universities raised from 31,000 to 7,909,900. And the proportion of higher education improved from 3.8% in 2000 to 19.4% in 2018. Considering the reality of China's huge population base, this trend not only reflects a simple increase in the proportion of advanced human capital, but also implies that China's labor factor advantage may change from “demographic dividend” to “human capital dividend” in the future. In contrast, the proportion of the higher education population in the United States reached 42% in 2010, indicating that there is still a big gap between China and developed countries in terms of high-level talents, and there is a long way to upgrade the human capital structure.

According to human capital theory, there is a strong correlation between health and education. The labor force with a lower education level tends to use health capital, so its health capital depreciation rate is higher and health level is lower (1). While a highly educated workforce not only accumulates more intellectual capital but also improves health capital, and has a higher level of health. The advanced human capital structure, which is mainly characterized by the rising proportion of human capital in higher education, may affect the level of public health. Clarifying the relationship between the advanced human capital structure and public health will help the government to carry out targeted intervention on public health, so this study is of great significance.

Previous scholars mainly study the impact of education on health in developed countries, while developing countries represented by China are quite different from developed countries in terms of economic development level, cultural background, and so on. Although China is the most populous country in the world, there is little literature on China. Therefore, selecting Chinese data to explore the relationship between advanced human capital structure and public health has important international comparison value. In addition, an in-depth discussion of the impact of human capital structure and its regional differences on public health will not only help to tap the potential of labor factors in China but also help to clarify the regional distribution characteristics of China's educational human capital structure. Thus, it provides a useful reference for establishing new educational human capital advantage and improving public health levels.

This paper takes into account the diminishing marginal effect of education human capital. Based on the health demand model of Grossman and panel threshold regression model, this paper mainly studies the relationship between advanced human capital structure and public health. The research focuses on: First, the advanced human capital structure is mainly manifested as the upgrading of educational human capital. Is there a clear causal relationship between advanced human capital structure and public health? If so, is there a non-linear relationship? Second, there are significant regional differences in China, so is there significant heterogeneity in the impact of advanced human capital structure on public health in different regions?

This paper has three main contributions. Firstly, the existing literature mainly demonstrates the causal relationship of education and health in developed countries, while this paper takes China as an example to explore the impact of advanced human capital structure on public health, which undoubtedly provides a beneficial supplement to the existing research. Secondly, this paper deeply analyzes the mechanism of the impact of advanced human capital structure on public health through the establishment of indicators for the advancement of human capital structure, based on the perspective of the dynamic evolution of human capital structure. Thirdly, considering the law of diminishing marginal effect of education human capital, the panel threshold regression model is used to discuss the non-linear effect of the advanced human capital structure on public health, and further explore the regional heterogeneity of the impact of the advanced human capital structure on regional public health.

The rest of the paper is organized as follows: Section “Literature review” reviews existing literature. Section “Health demand model of Grossman” introduces the health demand model. Section “Methodology and data” shows the data and introduces the panel threshold regression model. Section “Empirical Results” describes the empirical regression results and the robustness test results. Section “Conclusions” summarizes the research.

## Literature Review

Due to education and health as important components of human capital (2, 3), the relationship between education and health has attracted great attention in academic circles. Previous literature mainly studies from the following two aspects: Can higher levels of education affect public health? If so, what is the mechanism of action? In recent years, more and more scholars have paid attention to the impact of education on health (4–6). Most scholars believe that there is a strong correlation between education and health (3), and education can improve the level of health (7). On the one hand, the human capital of higher education has a better cognitive ability to its own health behaviors, such as reducing smoking, drinking and other behaviors, and increasing physical exercise (8), so as to maintain a healthy status of mind and improve the health self-assessment level (9). Adler et al. (10) and Hetzog et al. (11) also got a consistent conclusion that education can enable people to master the laws of mental health, maintain a good psychological status, and promote physical and mental health. On the other hand, high-quality talents generally have higher social status and better medical security, resulting in a lower mortality rate. For example, a study by Mazzonna (12) found that education could significantly improve the health of the elderly by improving the working environment and increasing income.

In addition, scholars have thoroughly studied the relationship between education and health in different countries and at different times (13–16). This conclusion has been confirmed whether the research object is the micro individual or macro whole (17, 18), whether using objective health indicators such as mortality or subjective health indicators such as self-rated health. Richards and Barry (19) showed that college students in the United States in 1990 lived more than 8 years longer than high school graduates. Kitagawa and Hauser (13), and Meara et al. (14) also reached a consistent conclusion. Mustard et al. (15) and Kunst and Mackenbach (16) selected the UK, Canada, and Northern Europe as research objects, and all believed that education could significantly improve health levels.

There are two hypotheses about the impact of education on health: “budget constraint relaxation theory” and “efficiency improvement theory.” The “budget constraint relaxation theory” believes that education can reduce the level of budget constraint on health input (20) and expand the budget constraint set of health input (7, 21). On the one hand, human capital with higher education can obtain higher social status and economic income by enriching their knowledge and skills, thus improving the nutritional conditions of their lives. On the other hand, high-level talents can make better use of medical and health information resources, which can improve the ability to benefit from advanced medical technologies by purchasing medical insurance, services, and health equipment (22). So, it is beneficial to the physical and mental health of the educated (20, 23). For example, Paul and Morris (24) studied the six health tracking data of 17,416 children born in 1958 in Britain, they found that education could significantly promote the health level of workers by improving their social status and improving their health behaviors. The “efficiency improvement theory” believes that, like the labor market, the labor force with higher education levels in the non-labor market can achieve relatively higher healthy production efficiency and resource allocation efficiency (3). First, under the given input factors, talents with higher education levels will bring higher health output (3), and maximize health production efficiency. For example, highly educated human capital can better cooperate with treatment through a comprehensive and in-depth understanding of the treatment plan, thus improving treatment effect (25). Second, the improvement of education level is conducive to improving the allocation efficiency of health input factors. In other words, people with higher education levels are better able to optimize their health investment portfolio (17, 26). Compared with the human capital of low education, the talents with higher education can understand the harm of bad living habits more clearly, and improve individual health conditions by adjusting healthy behavior. The improvement of education level can make individuals pay more attention to their health conditions. By reducing smoking, drinking, and other bad behaviors (27–29), cultivate good diet and exercise habits, improve self-management and control ability (6, 8, 20, 30, 31), and then improve their health level (32, 33).

The existing literature has many contributions: Firstly, scholars have conducted in-depth research on human capital and public health, but most scholars mainly study the impact of education on public health. There are few studies on the effect of education on public health from the perspective of dynamic change of human capital structure. Secondly, the existing literature in this field mainly focuses on the United States, Europe, and other developed countries. However, China and other developing countries have not been paid enough attention. Therefore, it is of great significance to research the relationship between advanced human capital structure and public health in developing countries taking China as an example. Thirdly, previous literature mainly studies the overall impact of education on health in a country or region, but regional differences are an important issue to study the advanced human capital structure on public health in China. In view of the huge differences in educational opportunities and quality among different regions, the internal relationship between the advanced human capital structure and public health level is likely to be affected. Therefore, this study also analyzes the regional differences in the impact of the advanced human capital structure on public health.

## Health Demand Model of Grossman

Grossman constructed a health demand model from the perspective of human capital, so this model is also known as the human capital model of health demand. Since the health production function constructed by Grossman is the mainstream theoretical basis of health research in the field of economics, this paper uses the Grossman model theory to analyze the mechanism of the advanced human capital structure on public health (3).

The Grossman model of health demand argues that, in contrast to education, health increases earning power mainly through increased working hours rather than productivity. Based on the human capital theory, Grossman first proposed the concept of health capital and made it clear that health capital was an important part of human capital by constructing a model of health demand.

Supposed that the utility function of a consumer in each period of his life is:


(1) $$\begin{array}{l} U=U\left(\varphi_{t}H_{t},Y_{t}\right),t=0,1,\cdots \;,n \end{array}$$

*H*_*t*_ represents the health capital stock which accumulated in *t* period, φ_*t*_ is the income of unit health capital, the health consumed in *t* period is *h*_*t*_ = φ_*t*_*H*_*t*_, and *Y*_*t*_ is equal to the number of other goods consumed in *t* period except for health. The initial health capital stock is *H*_0_, which is exogenous. *H*_*t*_ is endogenous in later stages and is chosen by consumers themselves. *n* represents the life of the consumer, and it's also endogenous. The increment of health capital is:


(2) $$\begin{array}{l} H_{t+1}-H_{t}=I_{t}-\delta_{t}H_{t} \end{array}$$

Where *I*_*t*_ expressions the investment in health capital in phase *t* and δ_*t*_ represents the depreciation rate. The depreciation rate is exogenous but varies with age. *I*_*t*_ and *Y*_*t*_ are determined by the following functions:


(3) $$\begin{array}{l} I_{t}=I_{t}\left(M_{t},TH_{t};E\right) \end{array}$$

(4) $$\begin{array}{l} Y_{t}=Y_{t}\left(N_{t},TY_{t};E\right) \end{array}$$

*M*_*t*_ refers to a series of goods that can be purchased, such as health services, which can be used as inputs to produce *I*_*t*_. *TH*_*t*_ denotes the time that spent improving health; *N*_*t*_ shows a general consumer product that can be purchased; the time used to produce *Y*_*t*_ is *TY*_*t*_. These four variables are endogenous. *E* represents human capital other than health, which is exogenous. The budget constraints that consumers face are:


(5) $$\begin{array}{l} \overset{n}{\underset{t=0}{\sum }}\frac{P_{t}M_{t}+Q_{t}X_{t}}{{\left(1+r\right)}^{t}}=\overset{n}{\underset{t=0}{\sum }}\frac{W_{t}TW_{t}}{{\left(1+r\right)}^{t}}+A_{0} \end{array}$$

*P*_*t*_ denotes the price of goods such as health services, *Q*_*t*_ is the price of general consumer goods, *W*_*t*_ is the wage rate, *TW*_*t*_ refers to the hours worked, and *A*_0_ represents initial wealth. In addition to budget constraints, consumers face time constraints. The total time in each period is Ω, and it has to run out in the current period, so


(6) $$\begin{array}{l} TW_{t}+TH_{t}+TY_{t}+TL_{t}=\Omega \end{array}$$

Where *TL*_*t*_ represents time lost because of poor health, such as time unable to work owing to illness. Equations 1–6 constitute the consumer's health demand model. The consumer's goal is to get maximum utility within budget constraints and time constraints.

Based on the above models, there are two ways to conduct empirical research on health demand: pure investment model and pure consumption model. Grossman (18) pointed out, “I emphasize the use of pure investment model rather than pure consumption model in empirical studies because the former has weaker assumptions and can generate strong predictions from the simple analysis.” Therefore, this research is also based on a pure investment model, and its equilibrium condition is:


(7) $$\begin{array}{l} \frac{G_{t}W_{t}}{\pi_{t-1}}+\frac{G_{t}\left[\left(U_{ht}/m\right){\left(1+r\right)}^{t}\right]}{\pi_{t-1}}=r+\delta_{t} \end{array}$$

$G_{t}=\frac{\partial TL_{t}}{\partial H_{t}}$ is equal to the marginal output of health, that is, the decrease of sick time brought by the increase of health level; $U_{ht}=\frac{\partial U}{\partial H_{t}}$ represents the marginal utility of health; *m* is the marginal utility of money income; π_*t*−1_ expressions the shadow price of health, which is determined by many factors, such as the price of health services and the wage level of consumers. The optimal condition (7) represents that marginal revenue equals marginal cost. Health income comes from two aspects: The first is the direct monetary income, namely $\frac{G_{t}W_{t}}{\pi_{t-1}}$, which is the same as the income of other investment products. The second is the utility directly brought by health, namely $\frac{G_{t}\left[\left(U_{ht}/m\right){\left(1+r\right)}^{t}\right]}{\pi_{t-1}}$. Like other capital goods, cost includes interest and depreciation.

**Figure 1:**
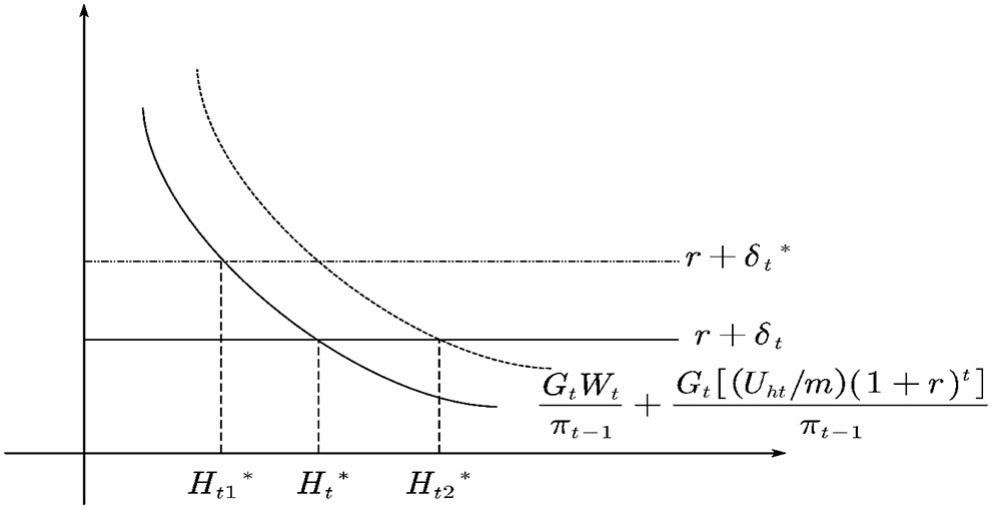
Comparative static analysis of health demand.

Equation 7 provides a series of testable theoretical predictions. As shown in Figure 1, the intersection of the health benefit curve and the cost curve, that's where $\left(\frac{G_{t}W_{t}}{\pi_{t-1}}+\frac{G_{t}\left[\left(U_{ht}/m\right){\left(1+r\right)}^{t}\right]}{\pi_{t-1}}\right)$ and (*r* + δ_*t*_) intersect, determines the optimal health demand, namely $H_{t}^{*}$. If the cost of investing in health increases, it leads to a decrease in the demand for health.

As for the variation of the depreciation rate, it is generally believed that the depreciation rate increases gradually with age. If the depreciation rate changes from δ_*t*_ to $\delta_{t}{\;}^{*}$, the demand for health will decrease from $H_{t}^{*}$ to $H_{t1}{\;}^{*}$. Education and health are complementary, and the advancement of human capital structure means that the proportion of human capital in higher education increases. This will increase the productivity of health human capital and reduce the shadow price of health. Thus, it causes the outward shift of the health income curve and increases the demand for health from $H_{t}^{*}$ to $H_{t2}{\;}^{*}$.

## Methodology and Data

### Methodology

#### Dynamic Panel Model

To verify the relationship between the advanced human capital structure and public health, this paper constructes an econometric model:


(8) $$\begin{array}{l} PH_{it}=\beta_{0}+\beta_{1}Hstruc_{it}+\beta_{2}CPI_{it}\\ +\beta_{3}PM2.5_{it}+\beta_{4}Density_{it}+\mu_{i}+\varepsilon_{it} \end{array}$$

Where *i* and *t* are the province and year; *PH*_*it*_ represents per capita health expenditure; the advanced human capital structure index is *Hstruc*_*it*_; *CPI*_*it*_, *PM*2.5_*it*_, and *Density*_*it*_ are consumer price index, *PM*2.5_*it*_ concentration in air and population density. β_0_ denotes a constant term; μ_*i*_ represents the unobserved regional effect, the error term is ε_*it*_. In addition, considering the continuity of *PH*, this paper adds a lag term of *PH* into Equation 8 and uses the dynamic panel model to perform the estimation test. The dynamic panel model not only reveals the dynamic variation characteristics of *PH* but also overcomes errors caused by the presence of endogeneity. The dynamic panel model established is set as follows:


(9) $$\begin{array}{l} PH_{it}=\beta_{0}+\beta_{1}PH_{it-1}+\beta_{2}Hstruc_{it}+\beta_{3}CPI_{it}\\ +\beta_{4}PM2.5_{it}+\beta_{5}Density_{it}+\mu_{i}+\varepsilon_{it} \end{array}$$

*PH*_*it*−1_ is the lag term of per capita health expenditure (*PH*). Other symbols have the same meaning as formula 8.

Since the independent variables of the empirical model include a lag term of *PH*, there may be a two-way causal relationship between the advanced human capital structure and per capita health expenditure (*PH*). In other words, the improvement of public health is in turn improve the level of advanced human capital structure, so the model maybe has endogenous problems. Using the Ordinary Least Square (OLS) method will lead to the error of model estimation, while the Dynamic GMM method can overcome the endogeneity problem. Difference GMM and System GMM are two important methods of Dynamic GMM estimation. Compared with the Difference GMM method, the System GMM method can solve the problem of weak instrumental variables and improve the estimation efficiency. Meanwhile, it can also estimate the coefficients of variables that do not change at any time. Considering that two-step GMM estimation may lead to deviation of the standard deviation of the estimated parameters, thus affecting the estimated results of parameters. So this paper adopts the one-step System GMM method to estimate the model.

#### Panel Threshold Regression Model

This paper considers the law of diminishing marginal effect of education human capital, the non-linear effect of advanced human capital structure on public health is discussed through the panel threshold regression model. The difference between a linear model and a non-linear model is whether there is an endogenous threshold variable. The PTRM model generally includes three steps: The first is to estimate the threshold value to ensure scientific results; The second is to classify the samples according to the threshold value; The third is to establish the relationship between explanatory variables and explained variables. This method can effectively eliminate the individual fixed effects, and the results are verified by using two-stage Ordinary Least Square. This paper uses the panel single threshold regression model of Hansen (34) to estimate the non-linear relationship between the advanced human capital structure and public health. {*PH*_*it*_, *Hstruc*_*it*_, *x*_*it*_:1 ≤ *i* ≤ *n*, 1 ≤ *t* ≤ *T*}, by establishing the following single threshold model:


(10) $$\begin{array}{l} PH_{it}=\{\begin{array}{l} \mu_{it}+\beta_{1}Hstruc_{it}+a_{1}^{'}x_{it}+\varepsilon_{it},if\;Hstruc_{it}\;⩽\;\gamma \\ \mu_{it}+\beta_{2}Hstruc_{it}+a_{2}^{'}x_{it}+\varepsilon_{it},if\;Hstruc_{it}>\gamma \end{array} \end{array}$$

Where *Hstruc*_*it*_ is the advanced human capital structure index as the threshold variable; *PH*_*it*_ is the per capita health expenditure, γ denotes the estimated threshold value; β_1_ and β_2_ are the threshold coefficients; the control variable is *x*_*it*_; $a_{1}^{'}$ and $a_{2}^{'}$ represent coefficients of the control variables; μ_*it*_ is the fixed effect in different provinces. ε_*it*_ denotes a white noise process compliance with $\varepsilon_{it}\sim \left(0,\;\sigma^{2}\right)$; *i* and *t* represent the provinces and time.

Equation 10 can also show as:


(11) $$\begin{array}{l} PH_{it}=\mu_{it}+\beta_{1}Hstruc_{it}\psi \left(Hstruc_{it}⩽\gamma \right)\\ \;+\beta_{2}Hstruc_{it}\psi \left(Hstruc_{it}>\gamma \right)+\alpha 'x_{it}+\varepsilon_{it} \end{array}$$

### Data

#### Variable Selection and Data Processing

Limited by data availability, this paper uses the data from China for 2009–2018. The data sources are the China National Bureau of Statistics, the China Statistical Yearbook, and the China Yearbook of Labor Statistics. We choose the advanced human capital structure index as the explanatory variable and the threshold variable. People with higher levels of education generally pay more attention to their health, so the advanced human capital structure can benefit public health (10, 11, 35). Per capita health expenditure (*PH*) as a public health indicator (36–38) As a major determinant of public health, health expenditure represents the level of health (39–41). Higher per capita health expenditure means higher levels of public health. Thus, per capita health expenditure is often used as a measure of public health.

This paper introduces three control variables. The first control variable is the consumer price index (*CPI*), which represents the changing trend in the price levels of consumer goods and services purchased by residents (42, 43). The level of *CPI* can explain the severity of inflation to a certain extent (44). Residents will measure the benefits and costs of health care expenditures to influence their health levels (45). The second is *PM2.5*, which affects public health by reducing air quality (46, 47). China's environmental pollution mainly comes from industrial emissions, and many studies show that the increase of air pollutant emissions has a significant impact on public health, so choose *PM2.5* as the air pollution indicator. Higher concentrations of fine particulate matter can increase mortality and a higher risk of death in areas with higher levels of industrial pollution (48). Finally, population density (*Density*) has also been adopted by many scholars as the main variable affecting public health. A larger population density can make health services more easily accessible to people under the constraints of a limited financial budget (49).

Table 1 is the descriptive statistics of variables. As can be seen from Table 1, the eastern region has the highest per capita health expenditure and is higher than the national average level. The central region is the lowest, at the same time both the central and western regions are lower than the national average level. This may be related to the higher level of economic development and medical in the eastern region of China. Similarly, the eastern region has the highest level of advanced human capital structure and is higher than the national average level. The western region is the lowest, both the central and western regions are lower than the national average level. On the one hand, the eastern region has developed economically and attached importance to education, so it has cultivated more human capital of higher education for the local area. On the other hand, the high level of economic development in the eastern region has attracted a large number of high-level talents from the central and western regions. However, owing to the relatively backward levels of economic development and education, the central and western regions have few high-level talents trained locally. And the two regions are not attractive to the higher education human capital from other regions.

**Table 1:** Descriptive statistics of the variables.

	**Variables**	**Mean**	**Std**.	**Min**	**Max**
China	*PH*	149.6400	86.5383	1.0000	299.0000
	*Hstruc*	18.16349	0.6890	16.5774	20.7350
	*CPI*	102.2947	1.5398	97.7000	106.3000
	*PM2.5*	47.7727	17.5999	15.7000	91.2000
	*Density*	4, 546.9200	2, 780.6190	557.000	12, 348.0000
Eastern China	*PH*	173.2000	89.6321	2.0000	298.0000
	*Hstruc*	18.6549	0.7677	17.4764	20.7350
	*CPI*	102.2691	1.5638	97.7000	106.1000
	*PM2.5*	50.1927	19.9605	15.7000	91.2000
	*Density*	5, 222.9360	3, 395.6240	864.000	12, 348.0000
Central China	*PH*	124.8000	77.6285	5.0000	299.0000
	*Hstruc*	18.1159	0.2850	17.2881	18.6920
	*CPI*	102.1987	1.4789	99.1000	105.8000
	*PM2.5*	52.2388	16.6110	20.8000	90.6000
	*Density*	5, 288.6250	2, 104.2560	2, 484.0000	9, 864.0000
Western China	*PH*	144.1455	84.2490	1.0000	297.0000
	*Hstruc*	17.7067	0.4450	16.5774	18.5425
	*CPI*	102.3900	1.5674	97.9000	106.3000
	*PM2.5*	42.1046	14.0824	17.8000	76.1000
	*Density*	3, 331.4820	2, 028.3260	557.000	8, 321.000

#### Measurement of the Advanced Human Capital Structure Index

This paper measures the advanced human capital structure by the method of space vector angle. The specific steps are as follows:

(1) Constructing space vector. According to the education level of employees, human capital is divided into the following five categories: illiterate and semi-illiterate, primary school, junior high school, high school, and college. And according to the space vector theory, taking the ratio that each type of human capital to the total amount of human capital as a component of the space vector. Then we construct a five-dimensional human capital space vector that contains five types of human capital *Y*_0_ = (*y*_0,1_, *y*_0,2_, *y*_0,3_, *y*_0,4_, *y*_0,5_).
(2) Selecting the reference vector, then measuring the angle between the space vector and the reference vector. If the ratio of a certain type of human capital to the total amount of human capital changes, the angle between the space vector and the reference vector will also change. Selecting the basic unit vector group *Y*_1_ = (1, 0, 0, 0, 0), *Y*_2_ = (0, 1, 0, 0, 0), *Y*_3_ = (0, 0, 1, 0, 0), *Y*_4_ = (0, 0, 0, 1, 0), *Y*_5_ = (0, 0, 0, 0, 1) as the reference vector, and calculating the angle between the human capital space vector and the reference vector:

(12) $$\begin{array}{l} \theta_{\text{m}}=arccos\left\{\frac{\overset{5}{\underset{\text{n}=1}{\sum }}\left(y_{m,n}\cdot y_{0,\text{n}}\right)}{{\left(\overset{5}{\underset{\text{n}=1}{\sum }}y_{\text{m},\text{n}}^{2}\right)}^{\frac{1}{2}}\cdot {\left(\overset{5}{\underset{\text{n}=1}{\sum }}y_{0,n}^{2}\right)}^{\frac{1}{2}}}\right\} \end{array}$$

In formula 12, *y*_m,n_ is the *n*th component of the *Y*_*m*_(*m* = 1, …, 5) which is the basic unit vector group, and *y*_0,*n*_ is the nth component of the vector *Y*_0_.

(3) Giving weight to the angle θ_*m*_, then weighted sum and obtain the advanced human capital structure index. The weight *V*_*m*_ of θ_*m*_(*m* = 1, …, 5) is determined by the coefficient of variation method: calculate *W*_*m*_ which is the coefficient of variation of θ_*m*_. Assuming that *TW* = *W*_1_ + … + *W*_5_, and *V*_*m*_ = *W*_*m*_/*TW*. In order to facilitate comparison with foreign data, setting *V*_1_, *V*_2_, *V*_3_, *V*_4_ and *V*_5_, which are the weights of θ_*m*_(*m* = 1, …, 5), in turn to 5, 4, 3, 2, and 1.

(13) $$\begin{array}{l} Hstruc=\overset{5}{\underset{m=1}{\sum }}\left(V_{m}\cdot \theta_{m}\right),V_{m}\text{ is the weight of }\theta_{\text{m}} \end{array}$$

*Hstruc* index comprehensively considers how relative changes of various human capital affect the advanced human capital structure (θ_*m*_). So *Hstruc* index reflects the overall level of the advanced human capital structure. According to the monotonic diminishing law of the inverse cosine function, in the process of continuous optimization of human capital, as the proportion of lower education human capital decreases, the proportion of higher education human capital will increase and θ_*m*_ will become larger. The larger *Hstruc* represents the higher the level of advanced human capital structure.

## Empirical Results

### Full Sample Empirical Results

In order to avoid pseudo-regression and ensure the stability of data, we use STATA16 to perform the LLC (50) tests and IPS teats (51). Table 2 shows that all variables are significant at the level of 5%. This means that there is no unit root in all variables. In other words, the data is stable and can be empirically tested by using the panel threshold regression model.

**Table 2:** Panel unit root tests.

**Variables**	**Panel augmented Dickey-Fuller test**			
	**Levin-Lin-Chu**		**Im-Pesaran-Shin**	
	***T*-statistic**	***P*-value**	***T*-statistic**	***P*-value**
*PH*	−29.1102^***^	0.0000	−7.1159^***^	0.0000
*Hstruc*	−13.8213^***^	0.0000	−2.9189^***^	0.0018
*CPI*	−6.2784^***^	0.0000	−5.9904^***^	0.0000
*PM2.5*	−19.5138^***^	0.0000	−6.8900^***^	0.0000
*Density*	−2.2820^**^	0.0112	−7.2159^***^	0.0000

***, and ** *respectively indicates significance at the 1, and 5% levels*.

In order to ensure the accuracy of the empirical results, this paper uses the one-step System GMM method to estimate the parameters of the constructed dynamic panel model. To eliminate the influence of heteroscedasticity on the model, robust standard error processing is adopted in this paper. Sargan's test of the model is significant at 1% level, representing that the tool variables selected are valid. Secondly, AR (2) of the second-order sequence correlation test shows that there is no autocorrelation problem, showing that the endogeneity problem of the model has been overcome. In addition, according to the study of Bond (52), in order to verify the effectiveness of one-step System GMM regression results, the FE method is adopted to estimate. As can be seen from Tables 3, 4, the estimation results of FE and GMM models show that the regression coefficient of advanced human capital structure is significantly positive at the level of 5%. It denotes that the advanced human capital structure can improve the public health level. And it is proved that the estimation results of one-step System GMM are valid. In the estimation results of the one-step System GMM, the regression coefficient of the *PH* lag term is significantly positive. Indicating that the *PH* is cumulative and persistent, further denoting that it is necessary to build a dynamic panel model for analysis in this paper.

**Table 3:** Full sample regression results of FE model.

**Variables**	**Coefficient**	**Std**.	***t*-value**	***p*-value**	**[95% conf. interval]**	
*Hstruc*	1.2290^**^	0.4787	2.57	0.016	0.2499	2.2081
*CPI*	−10.5892^***^	3.8078	−2.78	0.009	−18.3771	−2.8013
*PM2.5*	−0.0220^*^	0.0112	−1.96	0.059	−0.0450	0.0009
*Density*	0.0007^*^	0.0004	1.92	0.065	0.0000	0.0015
Cons	−9.0759	8.9654	−1.01	0.320	−27.4122	9.2604
R-squared	0.297

***, **, and * *respectively indicate significance at the 1, 5, and 10% levels*.

**Table 4:** Full sample regression results of GMM model.

**Variables**	**Coefficient**	**Std**.	***t*-value**	***p*-value**	**[95% conf. interval]**	
*L.PH*	0.4886^***^	0.1222	4.00	0.000	0.2491	0.7281
*Hstruc*	0.6915^**^	0.2989	2.31	0.021	0.1056	1.2774
*CPI*	−6.9842^**^	3.0772	−2.27	0.023	−13.0153	−0.9531
*PM2.5*	−0.0048	0.0078	−0.62	0.537	−0.0202	0.0105
*Density*	0.0003^***^	0.0001	3.40	0.001	0.0001	0.0001
Cons	−4.2282	5.1218	−0.83	0.409	−14.2667	5.8104

***, and ** *respectively indicate significance at the 1, and 5% levels*.

As can be seen from the estimation results in Tables 3, 4, the regression coefficient of *PH* is significantly positive. It can be concluded that since the implementation of the university enrollment expansion policy, the advanced human capital structure which is characterized by the increase in the proportion of higher education human capital has significantly improved the public health, and become a new driving force for the improvement of public health status. The possible reason is that the advanced human capital structure will raise the level of public health by improving residents' income level, social status, health awareness, medical level, and so on.

This paper uses the panel threshold regression model to test whether there is a non-linear relationship between advanced human capital structure and public health. Table 5 shows the self-sampling test of the advanced human capital structure threshold effect on public health. According to Hansen's threshold theory (34). In the single-threshold panel model, the *F* statistic is 28.45, and the corresponding *P*-value is 0.073. It means that at least a single threshold value is significant at the 10% confidence level. That is, the threshold variable of advanced human capital structure passes the single threshold test, and the threshold value is 20.3801. But the corresponding *P*-value is 0.2467 in the double-threshold panel model, so it failed the double threshold test. In a word, it proves that the model is non-linear (53–55).

**Table 5:** Threshold test results for the full sample.

**Threshold variable**	**Threshold**	**Threshold value**	**RSS**	**MSE**	***F*-stat**	**Prob**	**Crit10**	**Crit5**	**Crit1**
*Hstruc*	Single	20.3801	156.2530	0.5388	28.45^*^	0.0733	25.5444	31.3163	40.5886
	Double	20.3801	149.4781	0.5154	13.14	0.2467	17.7073	24.7319	46.8344

* *respectively indicate significance at the 10% levels*.

Table 6 presents the regression result of the relationship between advanced human capital structure and public health in China. The regression result shows that when the level of advanced human capital structure is lower than the threshold value of 20.3801, the regression coefficient of the advanced human capital structure index is 1.4951, and it is significantly positive at 1% level. It indicates that when the advanced human capital structure index is lower than the threshold value, it can significantly improve the public health status. When the level of advanced human capital structure is exceeded 20.3801, its regression coefficient is 1.3682, and it is significantly positive at 1% level, but the positive promoting effect is weakened. It can be seen that the impact of advanced human capital structure on public health shows a significant non-linear law of positive and diminishing marginal efficiency. The main reasons may be as follows: When the advanced level of human capital structure is lower, the advanced human capital structure gets rid of the constraints on the traditional labor force in knowledge and skills, and it provides strong support for talent cultivation, health perception, and technological innovation and so on. To be specific, first, the advanced human capital structure will improve the peoples' income level and social status, which will enable the public to invest more money in health, and then promote the improvement of public health. Second, the advanced structure of human capital can raise the awareness of their own health, maintain a healthy lifestyle, thus improving public health. Third, the advanced human capital structure will enhance the scientific and technological innovation ability of the society, and raise the social medical level by solving medical problems and developing high-tech medical equipment, which is conducive to the prolongation of life expectancy and the improvement of public health (56, 57).

**Table 6:** Panel threshold regression results for the full sample.

**Variables**	**Coefficient**	**Std**.	***t***	***P* > |*t*|**	**[95% conf. interval]**	
*CPI*	−11.5677^***^	3.2456	−3.56	0.000	−17.9581	−5.1773
*PM2.5*	−0.0217^***^	0.0068	−3.18	0.002	−0.0351	−0.0082
*Density*	0.0007^**^	0.0003	2.35	0.019	0.0001	0.0013
*Hstruc* (*Hstruc* ≤ 20.3801)	1.4951^***^	0.2200	6.79	0.000	1.0619	1.9284
*Hstruc* (*Hstruc* > 20.3801)	1.3682^***^	0.2155	6.35	0.000	0.9439	1.7925
_cons	−12.7872^***^	4.3109	−2.97	0.003	−21.2753	−4.2991

***, and ** *respectively indicate significance at the 1, and 5% levels*.

With the continuous improvement of human capital structure, the existence of information asymmetry may have a negative impact on the market to effectively guide the flow of high-end talents (58–60). When the labor market has not sent out the signal of demand-side saturation, the high-end talents still choose to go to areas where the industry is in urgent need of transformation and upgrading, thus leading to the situation of talent mismatch. It will lead to rising unemployment, social unrest, and other problems, and has a negative impact on public health.

In the sample period, most provinces don't cross the threshold value of the advanced human capital structure. It indicates that the promotion effect of the advanced human capital structure on public health is still limited. Under the background that China's labor factor advantage is changing from “demographic dividend” to “human capital dividend,” promoting public health by improving the level of advanced human capital structure is a powerful guarantee to achieve high-quality economic development. The regression results of the panel threshold are basically consistent with the FE model and GMM model. In this sense, the estimation results obtained by the panel threshold regression model are reliable.

### Regional Empirical Results

From a regional development perspective, the advanced human capital structure often presents obvious differences depending on the differences of regional factor resources, economic environment, and factor endowment. Therefore, this paper divides China's provinces into eastern, central, and western three regions to test the regional heterogeneity of the impact of advanced human capital structure on public health.

Table 7 shows the threshold test results for the eastern, central, and western regions. In eastern and central regions, the *P*-values of the single threshold test are 0.0167 and 0.0933, and the *F* statistics are 42.66 and 16.35. It represents that the eastern and central regions respectively pass the single threshold test at the level of 5 and 10%, and the thresholds value are 20.5972 and 17.9631. The results of the double threshold test show that the two regions do not pass the double threshold test. It indicates that there is a single threshold value of the advanced human capital structure index in the eastern and central regions. The *F* statistic and *P*-value of the western region are 19.35 and 0.1567, which indicates that the threshold test failed. That is, there is no threshold value in the western region.

**Table 7:** Threshold test results for the three regions.

**Threshold variable**	**Threshold value**	**Threshold**	**RSS**	**MSE**	***F*-stat**	**Prob**	**Crit10**	**Crit5**	**Crit1**
Eastern China	20.5972	Single	44.0470	0.4405	42.66^**^	0.0167	25.3358	31.7495	46.8407
	20.5972	Double	38.3523	0.3835	14.85	0.1667	37.9205	57.0513	105.7099
Central China	17.9631	Single	28.4556	0.4065	16.35^*^	0.0933	15.9246	19.0942	29.7647
	17.9631	Double	26.2462	0.3749	5.89	0.5433	13.3719	17.0367	22.1013
Western China	16.5789	Single	37.4860	0.3749	19.35	0.1567	23.8511	28.1527	48.4959

**, and * *respectively indicate significance at the 5, and 10% levels*.

Table 8 shows the panel threshold regression results of eastern, central, and western regions. It represents that advanced human capital structure can significantly improve the level of public health. But the promoting effect is different, the largest in the central region, followed by the western region and the eastern region is the smallest. In other words, there are obvious regional differences in the impact of the advanced human capital structure on public health. The reason may be that owing to China's vast territory, there are vast differences among regions, such as economic development level, technological level, resource endowment, and so on. However, advanced human capital structure is affected by institutional factors and other external factors. The human capital structure should be appropriate to the actual situation of each region to promote the improvement of public health levels. Therefore, only the human capital structure appropriate to the actual situation of each region, can it promote the improvement of public health (61–63).

**Table 8:** Panel threshold regression results for the three regions.

**Region**	**Variables**	**Coefficient**	**Std**.	***t***	***P* > |*t*|**	**[95% conf. interval]**	
Eastern China	*CPI*	−0.0003	0.0004	−0.73	0.466	−0.0012	0.0006
	*PM2.5*	0.0006	0.0104	0.06	0.956	−0.0201	0.0213
	*Density*	0.0013^***^	0.0003	4.18	0.000	0.0007	0.0020
	*Hstruc* (*Hstruc* ≤ 20.5972)	0.7344^**^	0.3112	2.36	0.020	0.1165	1.3522
	*Hstruc* (*Hstruc* > 20.5972)	0.5811^*^	0.3079	1.89	0.062	−0.0303	1.1925
	_cons	−12.4811^**^	6.1284	−2.04	0.045	−24.6491	−0.3130
Central China	*CPI*	−13.4891^**^	6.5241	−2.07	0.043	−26.5112	−0.4670
	*PM2.5*	−0.0395^***^	0.0116	−3.41	0.001	−0.0627	−0.0164
	*Density*	−0.0001	0.0009	−0.09	0.926	−0.0019	0.0017
	*Hstruc* (*Hstruc* ≤ 17.9631)	3.7814^***^	0.6318	5.99	0.000	2.5205	5.0424
	*Hstruc* (*Hstruc* > 17.9631)	3.7195^***^	0.6214	5.99	0.000	2.4793	4.9598
	_cons	−46.7924^***^	12.9328	−3.62	0.001	−72.6063	−20.9785
Western China	*CPI*	−11.9140^**^	5.1127	−2.33	0.022	−22.0653	−1.7627
	*PM2.5*	−0.0377^***^	0.0131	−2.88	0.005	−0.0637	−0.0117
	*Density*	0.0013	0.0012	1.06	0.291	−0.0011	0.0036
	*Hstruc*	1.4860^***^	0.3156	4.71	0.000	0.8594	2.1126
	_cons	−12.5273^*^	7.1954	−1.74	0.085	−26.8140	1.7594

***, **, and * *respectively indicate significance at the 1, 5, and 10% levels*.

In the eastern region, when the advanced human capital structure index is lower than the threshold value, the regression coefficient is 0.7344, which is significantly positive at 5%. While it exceeds the threshold value, the regression coefficient is 0.5811 and it is significantly positive at the 10% level. It means that the advanced human capital structure is conducive to improving public health, but the promotion effect will decrease when the threshold value is exceeded. That is, there is a non-linear relationship between them, which is consistent with the national test results. When advanced human capital structure exceeds the threshold value, the regression coefficient is 0.5811, which is significantly positive at the 10% level. The eastern region has diverse industrial distribution and a broad employment market, which is conducive to the gathering of senior talents, thus it has a great impact on the improvement of public health. However, the excessive concentration of senior talents will also lead to fierce competition in the job market, and then the rational allocation of human resources cannot be realized through normal signals of supply and demand in the labor market (64, 65). This will lead to an inefficient or invalid configuration of human capital, which will weaken the promoting effect of public health.

The central region passes the single threshold test, before and after the threshold value the regression coefficients of advanced human capital structure are significantly positive at the level of 1%. Specifically, the regression coefficient is 3.7814 when the advanced human capital structure is less than the threshold value, while the threshold variable exceeds the threshold value, its regression coefficient is 3.7195. It represents that advanced human capital structure has significantly improved the public health level in central China. However, because of the threshold effect, the promotion effect on public health when the advanced human capital structure index is less than the threshold value is greater than that when it is greater than the threshold value. When advanced human capital structure exceeds the threshold value, the promotion effect on public health will be weakened, which is consistent with the national test results. The possible reasons are as follows: The implementation of the strategy for the rise of central China creates conditions for the inflow of senior talents and advanced factors of production. And the central region is easier to obtain the spillover effect of human capital by virtue of its proximity to the eastern region. By learning, imitating, and innovating public medical policies and technologies from the eastern region, the region completes the local medical security system and raises the levels of medical care and residents' income, thereby improving the public health status. However, the excessive human capital structure will have some negative effects, and then reduce the promotion effect on public health.

In the western region, the regression coefficient of advanced human capital structure is 1.4860, and it is significantly positive at the 1% level, but there is no threshold effect. This indicates that advanced human capital structure can significantly promote the improvement of public health levels. With the policy guidance of the development strategy, the western region has attracted many high-level talents and advanced production factors which can improve the public health level. However, the economic foundation is weak and the talent introduction advantage is not strong, the future development of the western region still has a long way to go.

### Robustness Check

In order to ensure the accuracy of regression results, this paper selectes to add new control variables for the robustness test. The first new control variable selected in this paper is *GDP*. The Real *GDP* represents the level of economic development in a country or region. Economic growth can promote the improvement of social welfare and citizen income; thereby promoting public health (66–68). Another new control variable selected is mortality (*ML*), which is the ratio of the number of deaths to the average population during a period in an area. The mortality rate reflects the quality of health care in a certain area and it can significantly impact public health (69, 70). The constructed models are as follows:


(14) $$\begin{array}{l} PH_{it}=\mu_{it}+\beta_{1}Hstruc_{it}\left(Hstruc_{it}⩽\gamma \right)+\beta_{2}Hstruc_{it}\\ \;\left(Hstruc_{it}>\gamma \right)+a_{1}^{'}CPI_{it}+a_{2}^{'}PM2.5_{it}+a_{3}^{'}Density_{it}\\ \;+a_{4}^{'}GDP_{it} \end{array}$$

(15) $$\begin{array}{l} PH_{it}=\mu_{it}+\beta_{1}Hstruc_{it}\left(Hstruc_{it}⩽\gamma \right)+\beta_{2}Hstruc_{it}\\ \;\left(Hstruc_{it}>\gamma \right)+a_{1}^{'}CPI_{it}+a_{2}^{'}PM2.5_{it}+a_{3}^{'}Density_{it}\\ \;+a_{4}^{'}GDP_{it}+a_{5}^{'}ML_{it} \end{array}$$

As shown in Tables 9–12, after successively adding the new control variables, the regression results show that there is still a single threshold. It indicates that there is indeed a non-linear relationship between advanced human capital structure and public health in China. In conclusion, the robustness test results represent that advanced human capital structure is beneficial to the improvement of public health level, but there is a threshold value of the advanced human capital structure. When advanced human capital structure exceeds the threshold value, its promoting effect on public health will be weakened. This result is consistent with the previous one. It is proved that the regression results are robust and reliable (71).

**Table 9:** Threshold test results for the full sample after adding GDP.

**Threshold variable**	**Threshold**	**Threshold value**	**RSS**	**MSE**	***F*-stat**	**Prob**	**Crit10**	**Crit5**	**Crit1**
*Hstruc*	Single	20.3801	150.2197	0.5180	27.62^*^	0.0600	24.5840	28.6343	40.7452
	Double	20.3801	145.1258	0.5004	10.18	0.4200	19.5571	23.0538	41.2390

* *respectively indicate significance at the 10% levels*.

**Table 10:** Panel threshold regression results for the full sample after adding GDP.

**Variables**	**Coefficient**	**Std**.	***T***	***P* > |*t*|**	**[95% conf. interval]**	
*CPI*	−11.8556^***^	3.1896	−3.72	0.000	−18.1358	−5.5754
*PM2.5*	−0.0101	0.0076	−1.33	0.185	−0.0250	0.0048
*Density*	−0.0003	0.0004	−0.74	0.462	−0.0012	0.0005
*GDP*	0.0000^***^	0.0000	3.26	0.001	0.000	0.0001
*Hstruc* (*Hstruc* ≤ 20.3801)	1.1791^***^	0.2369	4.98	0.000	0.7126	1.6457
*Hstruc* (*Hstruc* > 20.3801)	1.0563^***^	0.2324	4.55	0.000	0.5988	1.5138
_cons	−3.4481	5.1147	−0.67	0.501	−13.5189	6.6227

*** *respectively indicate significance at the 1% levels*.

**Table 11:** Threshold test results for the full sample after adding GDP and ML.

**Threshold variable**	**Threshold**	**Threshold value**	**RSS**	**MSE**	***F*-stat**	**Prob**	**Crit10**	**Crit5**	**Crit1**
Hstruc	Single	20.3801	150.0459	0.5174	26.14^*^	0.0667	22.7002	28.5768	43.1279
	Double	20.3801	144.4162	0.4980	11.30	0.3700	19.2324	27.6444	37.1742

* *respectively indicate significance at the 10% levels*.

**Table 12:** Panel threshold regression results for the full sample after increasing GDP and ML.

**Variables**	**Coefficient**	**Std**.	***t***	***P* > |*t*|**	**[95% conf. interval]**	
*CPI*	−12.0265^***^	3.2087	−3.75	0.000	−18.3446	−5.7084
*PM2.5*	−0.0099	0.0076	−1.30	0.193	−0.0249	0.0051
*Density*	−0.0004	0.0005	−0.86	0.390	−0.0013	0.0005
*GDP*	0.0000^***^	0.0000	3.25	0.001	0.0000	0.0001
*ML*	−0.0883	0.1600	−0.55	0.581	−0.4034	0.2267
*Hstruc* (*Hstruc* ≤ 20.3801)	1.1806^***^	0.2373	4.98	0.000	0.7134	1.6478
*Hstruc* (*Hstruc* > 20.3801)	1.0598^***^	0.2328	4.55	0.000	0.6016	1.5181
_cons	−2.5054	5.3987	−0.46	0.643	−13.1356	8.1249

*** *respectively indicate significance at the 1% levels*.

## Conclusion

According to the health demand model of Grossman, this paper researches the relationship between advanced human capital structure and public health in China by using the panel threshold regression model. The conclusions are as follows: First, the advanced human capital structure can significantly improve the levels of public health, but there is a non-linear relationship between the advanced human capital structure and public health. When the level of the advanced human capital structure is lower than the threshold value, the promotion effect of advanced human capital structure on public health is greater. But when the advanced human capital structure index exceeds the threshold value, its promotion effect on public health will be weakened. Second, based on the results of the regional heterogeneity test, there are regional differences in the promotion effect of advanced human capital structure on public health. There is a threshold effect in the eastern and central regions, but there is no threshold effect in the western regions. At the same time, the central region has the greatest-promoting effect, while the eastern region has the lowest. Third, the results of the robustness test show that the regression results are robust and reliable. These conclusions can be used for reference in making medical policy in China. The labor departments should timely feedback market supply and demand signals, guide the rational allocation of human capital. At the same time, the governments should focus on the regional differences of advanced human capital structure and public health, and promote the coordinated development of regions.

## Data Availability Statement

The original contributions presented in the study are included in the article/supplementary material, further inquiries can be directed to the corresponding author.

## Author Contributions

T-HW: conceptualization, data curation, software, visualization, investigation, and writing—original draft preparation. JL: methodology and writing—reviewing and editing. Both authors contributed to the article and approved the submitted version.

## Funding

This research was partly supported by the National Social Science Fund of China (18BJL117).

## Conflict of Interest

The authors declare that the research was conducted in the absence of any commercial or financial relationships that could be construed as a potential conflict of interest.

## Publisher's Note

All claims expressed in this article are solely those of the authors and do not necessarily represent those of their affiliated organizations, or those of the publisher, the editors and the reviewers. Any product that may be evaluated in this article, or claim that may be made by its manufacturer, is not guaranteed or endorsed by the publisher.
